# Selecting the best candidate for a male incontinence device or another: dream or nightmare?

**DOI:** 10.1590/S1677-5538.IBJU.2020.0551.1

**Published:** 2021-02-03

**Authors:** Javier C. Angulo

**Affiliations:** 1 Universidad Europea de Madrid Hospital Universitario de Getafe Departamento Clínico Madrid Spain Departamento Clínico, Universidad Europea de Madrid, Servicio de Urología, Hospital Universitario de Getafe, Madrid, Spain.

## COMMENT

After initial encouraging results, the published outcomes of retrobulbar slings to treat post-prostatectomy incontinence can be considered suboptimal for moderate-to-severe incontinence, especially in patients with high body mass index and previous radiotherapy (1). However, the artificial urinary sphincter (AUS) is not the single alternative for these patients as different options of adjustable systems remain. The adjustable transobturator male system (ATOMS) has shown interesting results and can be an option even in radiated patients (2, 3). Other adjustable systems, such as male readjustment mechanical external system (male-REEMEX), adjustable sling Argus and adjustable continence therapy (ProACT) have different modes of action, variable results and also different security profile. Systematic reviews and meta-analysis allow indirect comparison of these systems, and the definition of their risk of failure, surgical revision and device explant (4–6). However, in the absence of randomized comparative studies the definition of the best patient profile for each device is not easy.

The aforementioned devices can be used to treat moderate-to-severe-incontinence in patients retaining certain sphincteric function. Then, the AUS is not the only resource to consider in a patient with stress incontinence other than mild. Besides, when natural history of the aging male, prone to manual dexterity and cognitive deterioration, is taken into account and the patient beyond the sphincter is fully considered (7) these options are very attractive, especially from the patients' point of view (8).

The standing cough test was proposed as a rapid and office-based surrogate for the evaluation of male postprostatectomy incontinence (9, 10). The male stress incontinence grading system (MSIGS) is a modified standardized application of the standing cough test was proposed to stratify patients according to leakage severity and to guide surgical planification. It quantifies standing cough test using a 0 to 4 scale based on the observed leakage pattern noted during four strong coughs in the standing position (11). It is true that the MSIGS correlates well to patient reported pad count (number of pads per day used) (11, 12), but a correlation with 24-h pad test has never been proved. Also, several limitations need being addressed. First of all, it is admitted that the patient has not voided at least 1 hour before the examination, but neither bladder filling needed for the assessment nor the intraabdominal pressure are standardized. Also, interobserver consistency of the test has never been evaluated and the MSIGS should be evaluated prospectively and not according to what is taken in clinical records.

Anyway, it is beyond discussion that the MSIGS can be of value to predict a negative result after male transobturator sling success and thus can be incorporated to refine predicted nomogram based on several independent variables that include (in order of decreasing weight) history of radiation, pad-count and MSIGS (12).

“Moderate male SUI: often worse than advertised” (13) searches for the optimal use of the MSIGS focusing on the population with moderate incontinence. The authors prove this population is composed of a varied group of patients, and in quite a big proportion of them severity of SUI could be higher than suspected. Unfortunately, due to the study design we only have data on the discrimination of two major groups (MSIGS 0-2 vs MSIGS 3-4), with a cut-off placed between MSIGS 2 (early drops, no stream with cough) and MSIGS 3 (drops initially, delayed stream). To my big concern this is the frontier in the “official literature” indication between sling (MSIGS 2) and AUS (MSIGS 3), while both cases are excellent indication for other devices (e.g., ATOMS). This article should not contribute to the establishment of an inaccurate paradigm based on the idea that male SUI can only be treated with an AUS (if severe) and retrobulbar sling (if mild), and that those with moderate incontinence can be segregated to one or the other group by the use of MSIGS.

Please do not forget a world of many other useful devices to treat male stress incontinence exist. Despite there is still a lot to investigate, moderate incontinence is a major group of patients and could be best treated with adjustable devices and not with retrobulbar slings (to reduce failure rate) or with AUS (to avoid significant overtreatment). Many surgeons worldwide successfully use other devices and that contributes to make more difficult the selection of the best candidate for a particular male incontinence device or another. Clinical trials and patient information will help to take the wise decision.
